# Evaluating the robustness of specialized and general-purpose facial expression recognition systems across varied scenarios

**DOI:** 10.3389/frai.2026.1800342

**Published:** 2026-08-17

**Authors:** José Salas-Cáceres, Javier Lorenzo-Navarro, Modesto Castrillón-Santana, Patricia Picazo-Peral, Sergio Moreno-Gil

**Affiliations:** 1 Instituto Universitario Sistemas Inteligentes y Aplicaciones Numricas en Ingeniera (SIANI), Universidad de Las Palmas de Gran Canaria, Las Palmas de Gran Canaria, Spain; 2 Instituto Universitario de Turismo y Desarrollo Sostenible (TIDES), Universidad de Las Palmas de Gran Canaria, Las Palmas de Gran Canaria, Spain

**Keywords:** affective computing, biometry, commercial software, FaceReader, facial expression recognition, validation, zero-shot learning

## Abstract

**Introduction:**

This work presents a comprehensive evaluation of facial expression recognition (FER) systems across four benchmarked datasets of varying complexity, ranging from controlled static images (ADFES, WSEFEP) to more realistic dynamic recordings (RAVDESS, CREMA-D).

**Methods:**

Three categories of models were evaluated: traditional FER neural networks models, general-purpose vision language models (VLMs), and the commercial software FaceReader^©^ 10.

**Results:**

The results show that performance on controlled datasets substantially overestimates real-world FER capability, with average weighted and unweighted average recall values decreasing from approximately 72% in static datasets to below 30% in naturalistic settings. All tested models exhibited a marked bias toward *happiness*, with negative emotions frequently misclassified, a trend particularly pronounced in VLMs, where categories such as *fear* or *anger* often received F1-scores near zero. Among the neural networks, the DAN model trained on the AfectNet dataset achieved the strongest generalization, outperforming all VLMs and confirming that AfectNet provides a more realistic training distribution than the RAF-DB database. FaceReader^©^ delivered excellent performance under ideal conditions but experienced substantial degradation in dynamic scenarios, falling below a random classifier in CREMA-D.

**Discussion:**

These findings highlight the limitations of general-purpose VLMs and commercial tools for real-world FER and underscore the need for models explicitly designed to handle naturalistic variability. Furthermore, the reported performance of FaceReader© 10 in their manual on ADFES and WSEFEP was corroborated in this study.

## 1 Introduction

In various practical fields, such as tourism and hospitality, understanding a users emotional response to a specific task or environment is a critical component for success (Manosso and Domareski Ruiz, 2021). Traditionally, researchers in these sectors have relied on questionnaires or customer reviews to gauge user satisfaction. While emotional expressions do not always represent a persons underlying internal state with absolute precision, they remain one of the most accessible and reliable proxies for obtaining a real-time estimation of human affect.

To move beyond the limitations of self-reporting bias where participants are consciously aware of being monitored, Facial Expression Recognition (FER) has emerged as a non-intrusive method for capturing spontaneous emotional data across multiple disciplines in real time (Canal et al., 2022). Nonetheless, they introduce significant technical challenges related to data acquisition, model generalization, and processing pipelines. To address these complexities, researchers opt to employ commercial integrated software solutions that claim to provide robust emotion analysis capabilities out of the box.

Several commercial platforms offer such solutions, including EmoVU by Eyeris Technology (Eyeris, 2025), AFFDEX 2.0 by Affectiva (iMotions, 2025), and FaceReader^©^ by Noldus (Noldus, 2025). These tools typically leverage visual data to estimate attributes such as emotional expression and soft biometric traits such as age group, and gender, among others. They are designed to operate without training, facilitating their deployment and use in real-world settings. Many of these solutions claim to achieve an almost perfect accuracy in detecting emotions (Noldus, 2025). However, these commercial systems are often trained on proprietary datasets and employ closed-source architectures, making independent validation and benchmarking difficult. Consequently, external evaluations must be performed using licensed software and standard public datasets, a task not always possible as some of the software are limited to not function in recorded data (Kramer, 2025).

Currently, the research in FER is shifting from classical approaches which rely on manually designed hand-crafted features paired with specific classifiers, toward more flexible architectures. Thus, foundational Large Language Models (LLMs) and Vision-Language Models (VLMs) are gaining attention due to their remarkable generalization capabilities (Marafioti et al., 2025; Chen et al., 2025). A key advantage of these models is their utility in zero-shot settings, where they can perform complex recognition tasks without task-specific training. Consequently, these foundational models are increasingly outperforming traditional neural networks that were specifically designed for narrower and specialized tasks. A recent example of this trend is the Pedestrian Attribute Recognition (PAR) contest, in which, across two consecutive editions (2023 and 2025), the winning approach was a zero-shot VLM that outperformed multiple task-specific neural networks trained directly for the problem (Castrillón-Santana et al., 2024; Greco and Vento, 2026).

It is worth noting that, in the machine learning community, FER remains an open research problem. Numerous state-of-the-art models have been proposed and benchmarked on publicly available datasets such as RAF-DB (Li and Deng, 2019), AffectNet (Mollahosseini et al., 2019), FER2013 (Goodfellow et al., 2015), RAVDESS (Livingstone and Russo, 2018), or SAVEE (Haq and Jackson, 2009). These datasets enable reproducible comparisons between models under standardized evaluation protocols.

Building on the discussion above, it is important to acknowledge that recent work challenges the long-held assumption that facial expressions directly reveal an underlying emotional state (Barrett et al., 2019). Evidence shows that similar facial configurations can communicate multiple meanings and vary substantially not only across individuals but also across cultures and contexts. Although a scowl may occur during anger, for example, it can also signal concentration or confusion, and many genuine emotional episodes do not involve the “stereotyped” facial patterns traditionally associated with them. Nonetheless, FER remains valuable because facial movements still convey rich social information that observers routinely use to infer intentions, interpersonal attitudes, and affective cues. Even without a strict one-to-one mapping between facial configurations and emotions, modeling these patterns allows FER systems to capture behavior that is highly relevant for human computer interaction and multimodal affective computing.

Based on this framework, in this paper a comprehensive comparison is conducted among three categories of models in a unified setting using standardized datasets. In addition to traditional neural networks, which rely on convolutional architectures or transformer-based visual encoders, the evaluation includes VLMs and a commercial software solution. The objective is to assess the generalization capabilities of these models and to compare them with a widely used commercial tool in related research fields. The commercial system selected for this study is FaceReader^©^ 10 by Noldus. This work is an extended version of the study presented in Salas-Cáceres et al. (2026), where additional VLM-based models are evaluated and two new datasets (ADFES and WSEFEP) are incorporated to enable a more comprehensive comparison across varied scenarios. Furthermore, the version of FaceReader^©^ used in the experiments has been updated from the 9th to the newer 10th release.

## 2 Related work

In the field of FER, a common distinction is made between *static* and *dynamic* approaches (Li and Deng, 2022). This differentiation is based on the temporal scope of the data processed by the model or presented on the dataset. *Static models* operate on individual frames, predicting the emotional state from a single, temporally isolated image. In contrast, *dynamic models* are designed to analyze sequences of frames, generating a single prediction by exploiting temporal information across an entire video segment. The same applies to databases, which portray single frames of an emotion in the static case or a whole sequence of the expression. Although the boundary between these two categories can be fluid, since dynamic datasets can be treated as static by processing each frame independently, it remains an important distinction, particularly when characterizing datasets. Dynamic datasets such as RAVDESS (Livingstone and Russo, 2018) or CREMA-D (Cao et al., 2014), typically provide richer contextual information, often including synchronized audio, speech transcripts, or motion cues, which are generally absent in static datasets. These additional modalities can be crucial for accurate emotion recognition, especially in realistic scenarios where multimodal approaches can lead to better performance due to the extra information they provide (Contreras-Higuera and Crescenzi-Lanna, 2025).

Another common categorization applied to FER datasets concerns the recording conditions under which the data were collected. In this regard, datasets are typically classified into two groups (Pan et al., 2023): *laboratory-controlled* datasets, such as RAVDESS (Livingstone and Russo, 2018), and *in-the-wild* datasets, such as MELD (Poria et al., 2019). The former are captured in highly controlled environments, where lighting, pose, and background are carefully managed to ensure optimal visibility and consistency across samples. These conditions facilitate the extraction of clear and unambiguous expressions. In contrast, in-the-wild datasets are collected under more naturalistic and unconstrained conditions, where variations in recordings are common. While these datasets better reflect the variability of real-world scenarios and often contain more authentic emotional expressions, they also introduce significant challenges for automated recognition systems due to the degraded or incomplete visual information.

As previously mentioned, in this paper the performance of the commercial software FaceReader^©^ 10 is evaluated, which, for internal validation, was evaluated on two publicly available datasets: the Amsterdam Dynamic Facial Expression Set (ADFES) (van der Schalk et al., 2011) and the Warsaw Set of Emotional Facial Expression Pictures (WSEFEP) (Olszanowski et al., 2015). According to the developers, the software achieved accuracies of 98.7% and 97.2% on ADFES and WSEFEP, respectively. These high performance levels on controlled datasets have been partially corroborated by external evaluations. In Küntzler et al. (2021), three commercial FER systems were tested on both highly standardized and in-the-wild datasets. The standardized set was constructed by aggregating four datasets, Karolinska Directed Emotional Faces (Lundqvist et al., 1998), ADFES, WSEFEP, and the Radboud Faces Database (Langner et al., 2010), while the in-the-wild set consisted of the Static Facial Expressions in the Wild (SFEW) dataset (Dhall et al., 2018). Their results highlighted a clear performance gap between controlled and real-world conditions. FaceReader^©^ 8, for instance, achieved up to 97% accuracy on the standardized dataset but only 31% on the SFEW dataset. Moreover, the study reported several issues related to face detection by FaceReader^©^ when applied to non-standardized or uncontrolled data. A broader evaluation of commercial FER tools was presented in Dupré et al. (2020), where eight systems were tested on a dataset comprising 937 videos from two sources: one capturing spontaneous expressions (UT-Dallas) and another with posed expressions (BU-4DFE). In that evaluation, accuracy scores ranged from 48% to 62%, with FaceReader^©^ 7 reaching a True Positive Rate (TPR) of 57.31%. Finally, in Kramer (2025), a comparative analysis between GPT-4o (OpenAI, 2024), a general-purpose large language model with multimodal capabilities, and FaceReader^©^ 7 was conducted using the WSEFEP dataset. The results indicated that GPT-4o outperformed the commercial software in the task of facial emotion recognition, even within this highly controlled and standardized environment. These studies suggest that, while commercial FER software such as FaceReader^©^ demonstrates high accuracy under ideal, controlled conditions, its generalization to more complex, real-world environments remains limited. This highlights the need for more extensive evaluations of such tools using dynamic and standard datasets.

## 3 Materials and methods

### 3.1 Datasets

This section presents a comprehensive overview of the datasets utilized in this study, detailing their origin, composition, and key characteristics relevant to the experimental design and analysis.

#### 3.1.1 WSEFEP

The Warsaw Set of Emotional Facial Expression Pictures (WSEFEP) is a visual dataset introduced in 2015 in Olszanowski et al. (2015). It contains images of 30 individuals displaying the six basic emotions defined by Ekman (1999): joy, anger, disgust, fear, surprise, and sadness, complemented by a seventh category: the neutral expression. The dataset was constructed through a highly controlled acquisition pipeline. Initially, 120 applicants participated in the selection process, which involved multiple stages, including interviews and preliminary photographic evaluations. Only 30 individuals satisfied the final inclusion criteria, which focused on acting experience, motivation, and the assessment of facial activity during the performance of the required expressions. The gender distribution was 16 females and 14 males.

**Figure 1:**
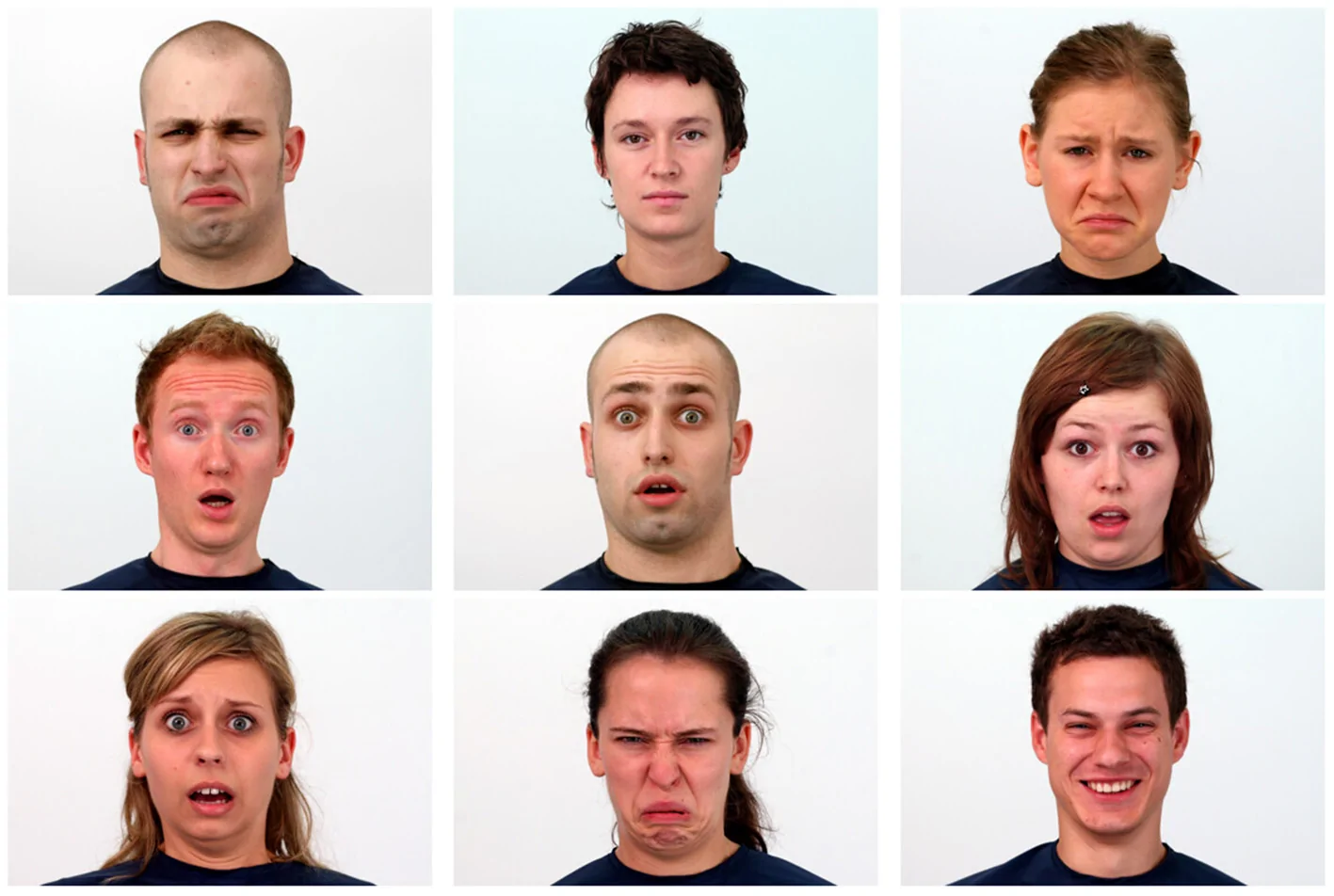
Some exemplary images of the WSEFEP dataset. Adapted from Olszanowski et al. (2015), licensed under CC BY 4.0.

For validation purposes, all images were evaluated by external and independent judges. Their ratings were used to determine the final subset of photographs according to the level of agreement on the emotions displayed and the judged intensity and purity of the expressed emotions, the higher, the better (Olszanowski et al., 2015). The resulting dataset comprises 210 images of Polish actors and is balanced across the seven expression categories. Representative examples are shown in Figure 1. It can be observed in the figure, the selection process yielded a demographically homogeneous group of actors, as all participants are Polish and Caucasian. The images also exhibit high levels of emotional intensity according to the criteria used during the selection procedure.

#### 3.1.2 ADFES

The Amsterdam Dynamic Facial Expression Set (ADFES) is a standardized collection of emotional facial expressions introduced in van der Schalk et al. (2011). It contains 648 recorded emotional expression sequences performed by 22 models (12 male, 10 female) from different ethnic backgrounds, originating from Northern Europe and the Mediterranean region, the latter including individuals with Turkish and Moroccan heritage. Despite these different backgrounds, all actors were Dutch-born, which limits the cultural diversity represented in the dataset. In addition to the full video sequences, the dataset provides apex still images extracted at the moment of maximal expressive intensity, which are commonly used in facial expression analysis research. The videos were validated by 119 undergraduate psychology students, who achieved an average recognition accuracy of 81% across the expression categories.

**Figure 2:**
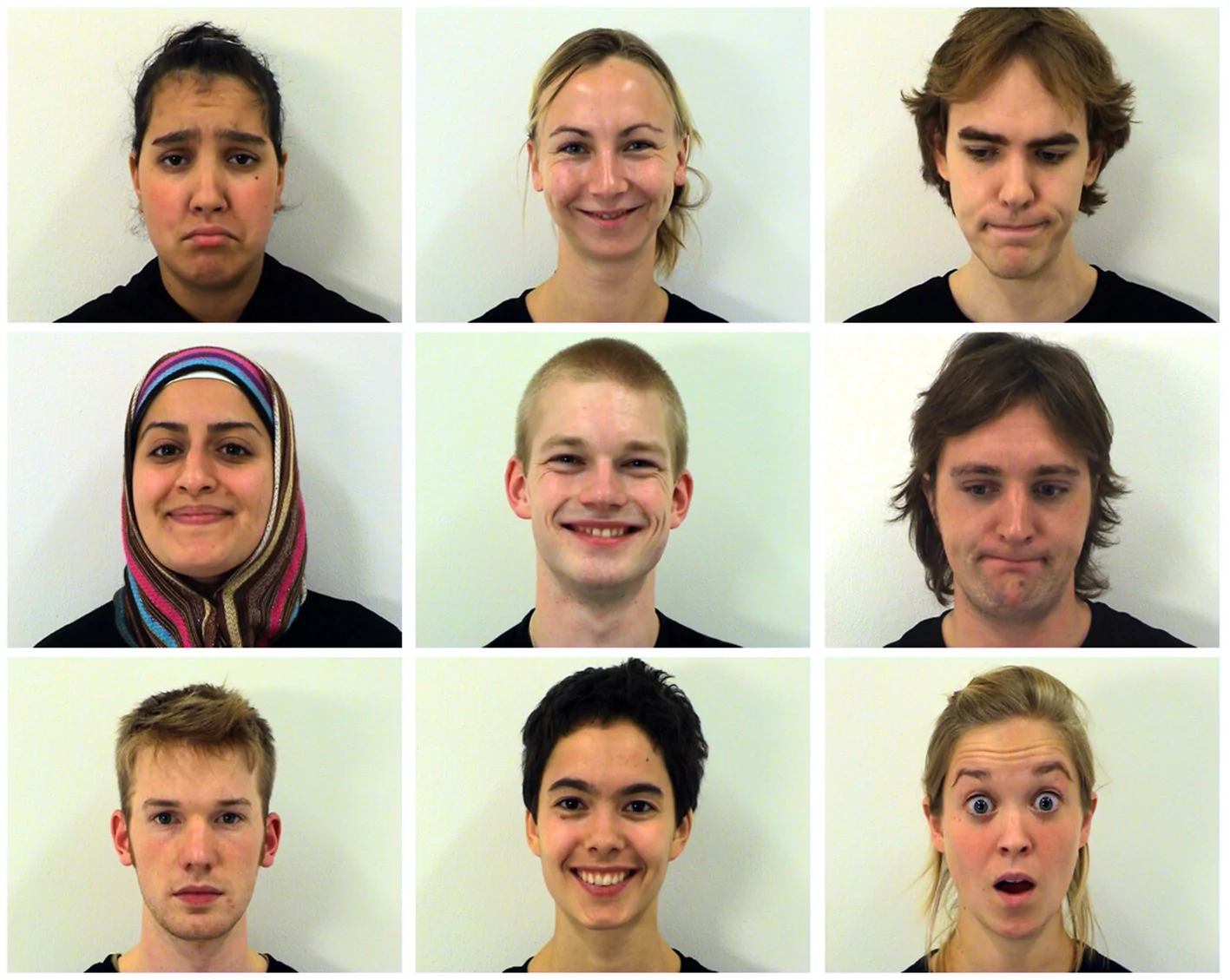
Some exemplary images of the ADFES dataset. Adapted from van der Schalk et al. (2011), licensed under CC BY 4.0.

A subsequent extension, the ADFES-Bath Intensity Variations (ADFES-BIV), was introduced in Wingenbach et al. (2016). This version includes video sequences captured at multiple intensity levels for each emotion. Figure 2 presents several apex frames extracted from the dataset. Similar to the WSEFEP dataset, the expressions in both ADFES and ADFES-BIV display high emotional intensity according to the criteria applied during their construction. Although the ADFES-BIV provides additional granularity, only the apex frames of the original ADFES dataset are used in this study, as this is the dataset specified in the FaceReader^©^ 10 manual (Noldus Information Technology, 2025).

#### 3.1.3 RAVDESS

The Ryerson Audio-Visual Database of Emotional Speech and Song (RAVDESS) is an audiovisual dataset first introduced in Livingstone and Russo (2018). It comprises recordings of 24 professional actors (12 male and 12 female) performing eight emotional states: neutral, calm, happy, sad, angry, fearful, disgusted, and surprised. The dataset contains a total of 7,356 recordings, with each actor contributing 104 unique clips. Each emotion was portrayed twice, once with normal intensity and once with heightened intensity, except for the neutral emotion, which was recorded only once per actor. The dataset is well-balanced in terms of gender representation, however, there is a predominance of Caucasian individuals, with limited representation of other ethnic groups. RAVDESS is divided into two subsets: one in which actors sing, and another in which they speak. For the purposes of this study, only the speech subset was used, as it more closely resembles realistic interactive scenarios.

**Figure 3:**
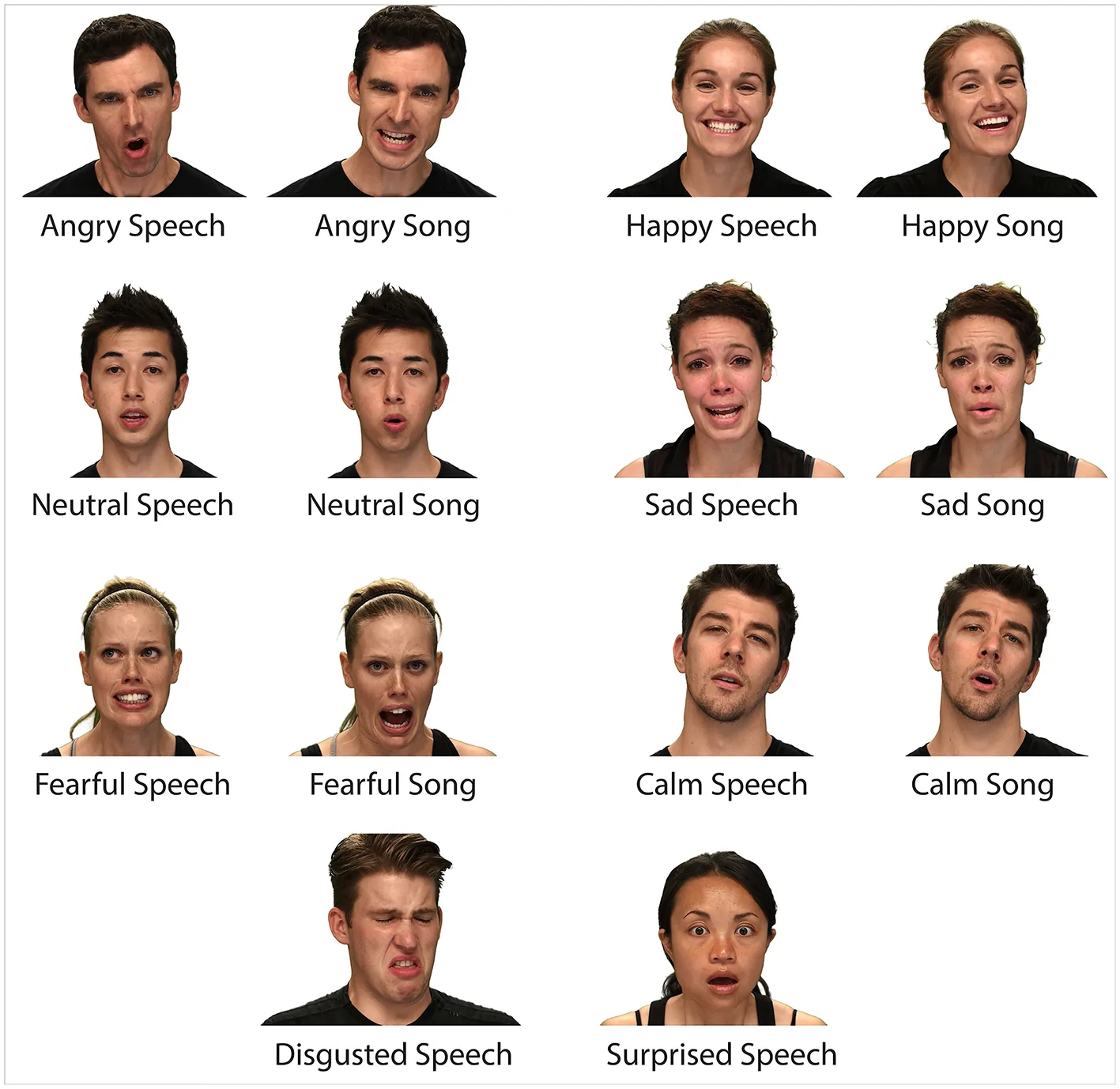
Example of frames seen in RAVDESS. Extracted from Livingstone and Russo (2018), licensed under CC BY 4.0.

As mentioned previously, each emotion was expressed at two different intensity levels. In this study, no distinction was made between the two meaning both were treated as equivalent instances of the same emotional category. All recordings were captured in a professional studio environment, under controlled lighting conditions, using high-quality cameras and microphones, lasting between 3 and 5.5 seconds. Representative frames illustrating the overall visual quality of the dataset are shown in Figure 3. RAVDESS is widely used and recognized within the affective computing community, as highlighted in Luna-Jiménez et al. (2022).

#### 3.1.4 CREMA-D

The Crowd-sourced Emotional Multimodal Actors Dataset (CREMA-D) is a comprehensive audiovisual corpus comprising 7,442 clips performed by 91 actors, 48 male and 43 female, as described in Cao et al. (2014). The dataset demonstrates an intentional effort to balance actor demographics in terms of gender, age, and ethnicity.

Each actor was instructed to articulate a predefined set of 12 sentences designed to elicit a diverse range of emotional expressions. The dataset includes six emotion categories, each portrayed at four distinct intensity levels. While CREMA-D provides annotations for emotional intensity, the present study focuses solely on emotion classification. All intensity levels were treated equally, given their relatively uniform distribution across samples.

**Figure 4:**
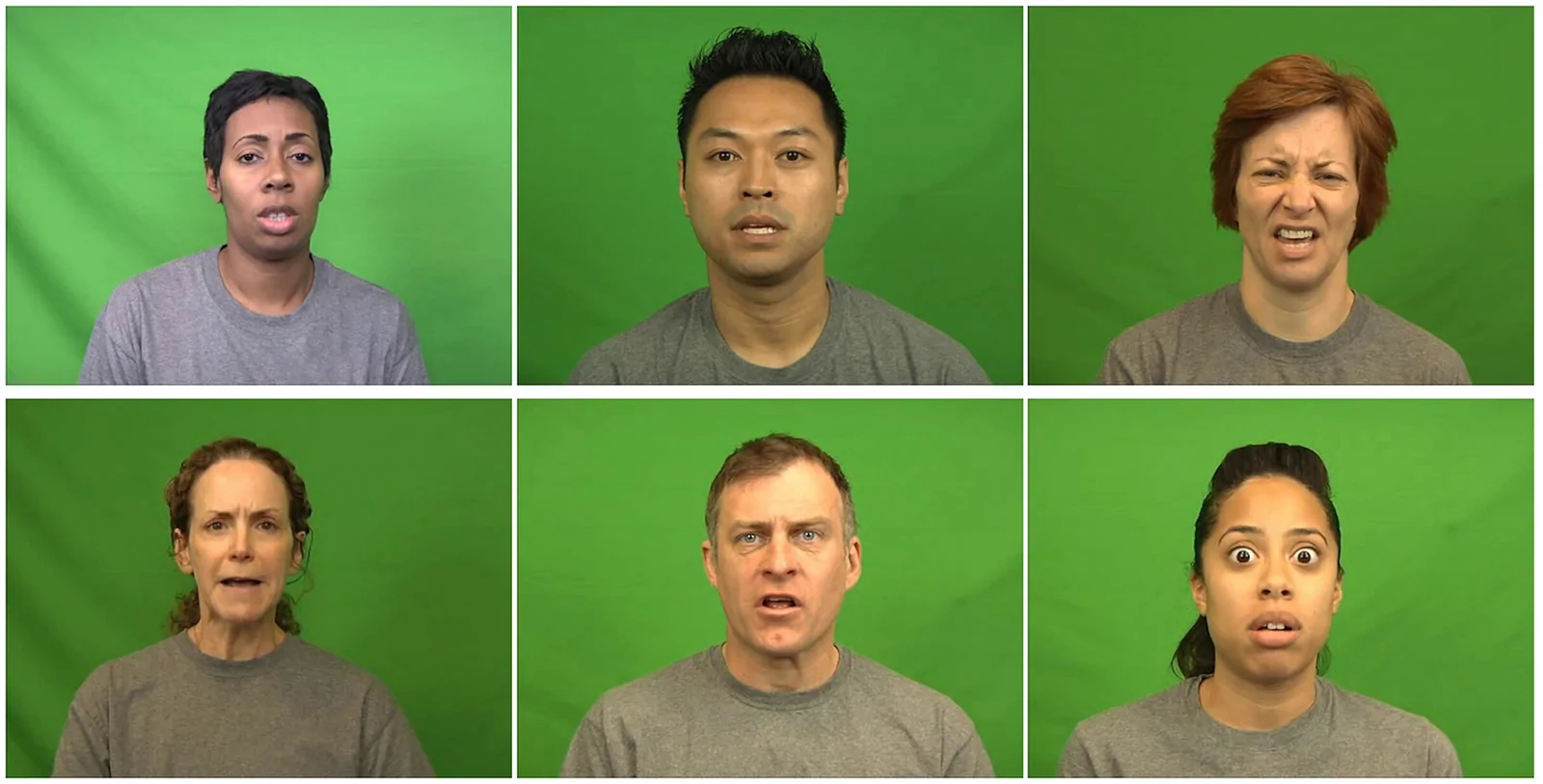
Example of frames seen in CREMA-D. Adapted from Cao et al. (2014), licensed under the Open Database License.

Although the recordings were conducted under controlled conditions, the overall visual quality of the videos is somewhat limited by the camera's native resolution of 960 × 760 pixels. Representative frames illustrating this quality are presented in Figure 4.

In Table 1, a comparison of the four datasets used in this study is presented. The selection was motivated by two factors. First, ADFES and WSEFEP were chosen to validate the accuracy reported in the FaceReader^©^ manual (Noldus Information Technology, 2021). Second, to evaluate the software on datasets not included in its commercial training data, two dynamic, and therefore unseen, datasets were selected, CREMA-D and RAVDESS. These two datasets, aside from being widely used in the literature, contain expressions of lower intensity and exhibit a more natural display of emotions. By using these four different datasets, the models can be evaluated across a broad range of scenarios, ensuring a more comprehensive assessment of their performance.

**Table 1:** Dataset comparison.

Dataset	Number of individuals	Temporal data	Resolution (pixels)	Published year
WSEFEP	30	No	1,725 × 1,168	2015
ADFES	22	Yes	720 × 576	2011
RAVDESS	24	Yes	1,280 × 720	2018
CREMA-D	91	Yes	960 × 760	2014

### 3.2 Evaluated models

In this section, the FER models evaluated in this study are briefly introduced and defined, with emphasis placed on their specific characteristics and their respective approaches to the FER task. It should be noted that FaceReader^©^ is not included in this subsection, as it has already been described in Sections 1, 2 of this work.

#### 3.2.1 Traditional neural networks

The first subset of tested models are traditional neural networks trained on FER specific dataset.

- **DAN**: The Distract Your Attention Network (DAN) proposed by Wen et al. (2023) is a FER model designed to distinguish subtle, visually similar expressions while capturing information from multiple facial regions simultaneously. Its architecture consists of three main modules: a Feature Clustering Network that extracts robust features and is trained to maximize class separability; a Multi-head Attention Network that attends to several facial areas in parallel; and an Attention Fusion Network that merges these attention outputs and also serves as the classification head.
- **SwinFace**: SwinFace, introduced by Qin et al. (2024), is a facial analysis model built upon Swin Transformers, a variant of Vision Transformers (ViT) by Dosovitskiy et al. (2021). It constist of an encoder that is then followed by multiple task-specific sub-classifiers dedicated to different tasks such as facial expression recognition, gender classification, age estimation, among others.
- **ResMasknet**: ResMaskNet, proposed by Pham et al. (2021), centers on the use of residual connections through four Residual Masking Blocks, each one composed of 2D convolutional layers with residual links followed by a masking module based on a U-Net–like encoder–decoder architecture.
- **AP-ViT**: Based on the ViT architecture, AP-ViT, developed by Xue et al. (2023), modifies the original design by applying patching not to the raw image but to the feature maps produced by an encoder. A convolutional backbone such as ResNet first extracts these feature maps, from which an attention map is generated. This attention map guides the selection of the most informative feature patches. The chosen patches are then flattened, prepended with a classification token, and fed into a multi-head attention module that highlights the most discriminative tokens, ending with a classification based only on the previously added token.

#### 3.2.2 Vision-language models

The second subset of models consists of VLMs, which require a textual prompt to operate and are typically composed of a visual encoder coupled with a language model.

- **SmolVLM**: Developed by the team behind Hugging Face, SmolVLM is a compact VLM built by pairing the smallest variants of the SigLIP family of encoders proposed by Zhai et al. (2023) with the lightweight SmolLM2 language model introduced by Allal et al. (2025). This architecture emphasizes coupling smaller versions of both visual encoder and language models, architecture emphasizes tight coupling between a modest-sized vision encoder and a small LLM, as the authors observe that using a disproportionately large vision encoder with a small language model degrades overall performance. Based on this, SmolVLM is designed to compete in performance with the bigger VLMs by maintaining a modest size as demonstrated in Marafioti et al. (2025).
- **FaceLLM**: In general, VLMs are designed as general-purpose models and are explicitly trained to avoid overfitting to a single task. Despite this broad focus, they often achieve strong performance across a wide range of domains. In contrast, FaceLLM is a model derived from Zhu et al. (2025) and is trained specifically for facial attribute recognition. Owing to this task-oriented training, Shahreza and Marcel (2025) demonstrated that the model exhibits superior performance in biometric applications when compared with more general VLMs.
- **PaliGemma**: Both PaliGemma 1 and PaliGemma 2 combine the SigLIP encoder with Googles Gemma language model, first published by Team et al. (2024), to form compact visual–language models (fewer than 3B parameters) while maintaining competitive performance as illustrated in Beyer et al. (2024); Steiner et al. (2024). In this study, the 3B-mix-224 variants of both models were evaluated.
- **BLIP-2**: The BLIP-2 model, introduced by Li et al. (2023), employs a modular architecture that integrates two pretrained components: a visual encoder, such as a ViT, and a large language model (LLM). These components are connected via a querying transformer that bridges the modality gap. Training proceeds in two stages: first, the frozen visual encoder is bootstrapped, followed by a similar procedure applied to the LLM. This approach produces a multimodal model capable of strong performance while requiring significantly fewer parameters than conventional architectures.

### 3.3 Methodology

This section describes the procedure used to evaluate all models considered in this study and introduces the metrics employed in the assessment.

#### 3.3.1 Evaluation pipeline

**Figure 5:**
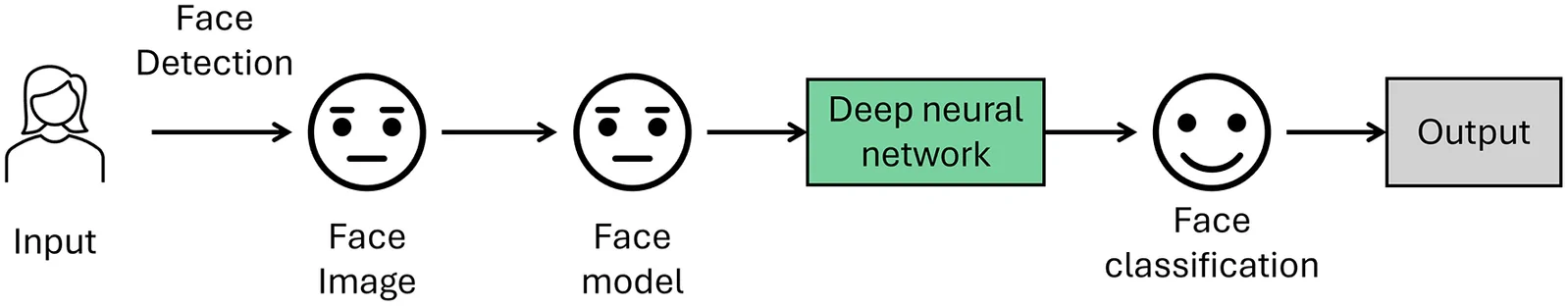
Facereader^©^ 10 pipeline to achieve emotion classification. Information extracted from the User manual (Noldus Information Technology, 2025).

As it can be seen in Figure 5, the evaluation pipeline applied to each model follows a structure commonly employed in biometric and affective computing systems, which is also implemented in the commercial software considered in this study. This pipeline consists of three main stages:

1. **Face detection and alignment**: The initial stage involves detecting and aligning the face within each frame. For the experimental setup presented in this study, face detection was performed using RetinaFace (Deng et al., 2019) on all datasets to ensure consistent input across models. In contrast, FaceReader^©^ employs its proprietary face detection module (Zafeiriou et al., 2015; Noldus Information Technology, 2021; Loijens and Krips, 2018; Noldus Information Technology, 2025). This module has limitations and tends to fail in certain situations where lighting conditions and image quality are suboptimal (Küntzler et al., 2021; Burgess et al., 2023). Because of the impossibility of bypassing this module when using FaceReader^©^, RetinaFace was not applied when testing on it. However, due to the controlled nature of the environments in which the datasets were recorded, this is not expected to pose a significant issue.
2. **Face modeling**: In the second stage, a feature representation of each face is generated. The approach to face representation varies across models; some models compute embeddings using other architectures like DeepFace (Serengil and Ozpinar, 2024) or VGG face (Cheng and Zhou, 2020), while others operate directly on raw pixel data. FaceReader^©^ utilizes a deep neural network described in Gudi et al. (2015) to generate a synthetic representation of the face, extracting 468 facial landmarks. Subsequently, Principal Component Analysis (PCA) is applied to reduce the dimensionality of this representation (Noldus Information Technology, 2021; Loijens and Krips, 2018). In line with standard Transformer designs, VLMs rely on an internal encoder to project visual data into a latent space. For instance, SmolVLM (Marafioti et al., 2025) utilizes the SigLIP encoder to generate facial representations, effectively bridging the visual input with the models internal language module in a manner consistent with traditional network architectures.
3. **Facial expression recognition**: In the final stage, emotion classification is performed using either the extracted embeddings or the original pixel-based input, depending on the model. Although FaceReader^©^ applies PCA to the set of facial landmarks, it is reported that classification is ultimately performed on the facial image itself, following the methodology outlined in Gudi et al. (2015). In all models evaluated in this study, the classification task involves recognizing the six basic emotions defined by Ekman in Ekman (1999), in addition to the neutral expression. When working with VLMs, this step requires a prompt that specifies the task to be performed by the multimodal model.

Although the evaluation pipeline was consistent across all models, each model category required specific implementation details:

- For FaceReader^©^, which natively operates on still images, the ADFES and WSEFEP datasets were processed directly by inserting all images of each dataset into a single software individual, generating predictions in a single file for each. For the dynamic datasets, RAVDESS and CREMA-D, a separate project was created, as it is not possible to work at the same time with still images and videos. To simplify processing, all videos were concatenated into a single file per project. In this case, the program's output consisted of a file for each video, containing predictions for every frame, enabling subsequent frame-level analyses.
- For traditional neural networks, a similar approach was followed, as all tested models were capable of processing only still images. Predictions were computed for each image in the ADFES and WSEFEP datasets. For the CREMA-D and RAVDESS datasets, individual frames were extracted from the concatenated videos and processed independently. Similarly to the previous case, the concatenation process does not alter the behavior of these models, as predictions are performed at the frame level, allowing each sample to be processed independently.
- For VLMs, the same general pipeline was applied. In this case, a task-specific prompt was required to instruct the model to perform facial expression recognition.Following a similar approach as in Salas-Cáceres et al. (2025), several prompts were evaluated to optimize model behavior. Each prompt employed different wording and slightly varied instructions for the visual model, while maintaining a common constraint requiring the response to consist of a single word. The set of prompts considered in this study is presented in Table 2. Because VLMs do not produce responses from a fixed set of classes, a dictionary of synonyms was employed to standardize outputs. In the rare cases that the output do not correspond to any of the emotions, i.e., the model just responds “no,” “yes,” or “unanswerable,” the prediction was deemed invalid and therefore an error. The dictionary used to map model outputs to the fixed set of emotion categories is presented in Table 3.

**Table 2:** Different prompts tested.

Prompt name	Prompt
Q1	*In a single word, is the person angry, disgusted, happy, sad, fearful, surprised or neutral? Do not add any additional information*.
Q2	*What is the emotion of the person?, use just one word to answer*
Q3	*Using only one of the basic emotions (anger, disgust, happy, sad, fearful, surprised or neutral), what emotion is the person expressing?. Use just one word*.
Q4	*The image shows the face of a person expressing a single emotion among the basic emotions (anger, disgust, happy, sad, fearful, surprised, or neutral). Which emotion is it? Answer with just one word*.
Q5	*What emotion does the image evoke? Use just one word to answer*.

**Table 3:** Dictionary used to map model outputs to the fixed set of basic emotions.

Emotion class	Synonyms
Anger	Angry, sullen, anger, frown, mad, stern, annoyed, pained, irritated, frustration, frustrated, and annoyance
Disgust	Disgust, disgusted, disgusting, sour, disapproval, displeasure, contempt, and contemptuous
Fear	Fearful, frightened, fear, afraid, afraidness, scared, worry, nervousness, worried, suspense, suspicious, suspicion, uncomfortable, and concern
Happiness	Happy, pride, proud, prideful, smug, happiness, joy, excited, joyful, smiling, nodding, smile, quizzical, excitement, smiles, humor, smiley, amusement, amuse, satisfied, satisfaction, and laugh
Neutral	Neutral, calm, calmness, peaceful, relaxed, content, serene, composed, peace, embarrass, embarrassed, embarrassment, bored, boredom, confusion, curiosity, pensive, sarcastic, determined, determination, confused, serious, squinting, expressionless, still, contemplative, blank, white, interest, and concentration
Sad	Sad, sadness, dissatisfied, sobbing, crying, disappointed, depressed, pain, gruff, displeased, sorrow, and sorry
Surprise	Surprised, surprise, astonished, astonishment, shock, and shocked

It is important to note that the proposed experiments constitute a zero-shot setting for the VLMs, but not for the traditional FER models or the commercial solution. Strictly speaking, zero-shot classification requires inference on a label space that differs from the one used during training (Pourpanah et al., 2023), whereas the traditional models and FaceReader^©^ were originally developed to recognize the same facial expressions present in the evaluated datasets. Consequently, for these systems, the experimental setup is more appropriately described as a cross-dataset generalization evaluation, where performance is assessed on datasets that differ from those used during development or training.

#### 3.3.2 Evaluation metrics

For the ADFES and WSEFEP datasets, the evaluation pipeline concludes once all image-level predictions have been collected. In contrast, a post-processing step is required for the CREMA-D and RAVDESS datasets, since the tested models operate without temporal modeling capabilities while both datasets consists of video samples. To obtain a single prediction per clip, a voting scheme was applied in which the assigned emotion corresponds to the class occurring most frequently within the temporal boundaries of each video. Because the videos were concatenated for easier processing on the FaceReader^©^ software, predictions whose temporal span exceeded the duration of the corresponding sample were truncated to its time interval. This procedure ensures that a single, video-level prediction is produced for each clip.

As evaluation metris, in the FER literature, performance is often reported using *Unweighted Average Recall* (UAR) and *Weighted Average Recall* (WAR). UAR represents the mean recall across all classes, *Recall*_*i*_, without considering the number of samples per class. In contrast, WAR computes a recall average weighted by the number of true instances per class *i*_*th*_, *N*_*i*_, relative to the total number of samples, *N*_*total*_, making it more suitable for evaluating model performance on imbalanced datasets. *TP*_*i*_ and *FN*_*i*_ refer respectively to the number of true positives and false negatives of class *i*.


$$\text{UAR}=\frac{1}{C}\overset{C}{\underset{i=1}{\sum }}{\text{Recall}}_{i}\;\text{where}\;{\text{Recall}}_{i}=\frac{TP_{i}}{TP_{i}+FN_{i}}$$

$$\text{WAR}=\overset{C}{\underset{i=1}{\sum }}w_{i}\cdot {\text{Recall}}_{i}\;\text{where}\;w_{i}=\frac{N_{i}}{N_{\text{total}}}$$

For each individual emotion, the F1-Score, as defined in Equation 1, is also reported. This metric provides a balanced assessment of precision and recall, giving an understanding of how well the model performs at predicting a given class.


$$\begin{array}{l} \text{F1-Score}=2*\frac{{\text{Recall}}_{i}*{\text{Precision}}_{i}}{{\text{Recall}}_{i}+{\text{Precision}}_{i}} \end{array}$$ (1)

$$\begin{array}{l} \;\text{where}\;{\text{Precision}}_{i}=\frac{TP_{i}}{Tp_{i}+FP_{i}} \end{array}$$

## 4 Results and discussion

This section presents the results obtained by the tested models on each one of the four datasets. For the VLMs, the results presented correspond to the best-performing question in terms of WAR. Each table will also contain the 95% confidence intervals of the metrics. The impact of the different prompts on model performance is analyzed in Section 4.4. Before presenting the dataset results, a brief analysis is carried out to assess the impact of face detection failures.

### 4.1 Face detection analysis

As explained in Section 3.3.1, the first stage of the FER pipeline consists of face detection and alignment. Consequently, failures at this stage are expected to propagate through the remainder of the pipeline, often resulting in incorrect emotion predictions, as the evaluated models are designed to operate on facial images. To minimize the impact of face detection on the comparison, the same detector, RetinaFace, was employed for almost all evaluated models, ensuring that any detection errors would be shared across methods. An exception is FaceReader^©^, whose internal face detection module is neither publicly documented nor configurable by the user. Furthermore, FaceReader^©^ generates an emotion prediction regardless of whether a face has been successfully detected, making a direct assessment of its detection performance impossible. To estimate the potential influence of face detection failures on the reported results, an additional analysis was conducted using both RetinaFace and the classical Viola-Jones Haar Cascade detector (Viola and Jones, 2001), a simple and widely used face detection method. The selection of this algorithm was also motivated by the FaceReader^©^ documentation, which indicates that the software relies on a face detection algorithm that is “more accurate, robust, and faster” than the Viola-Jones Haar Cascade detector. Therefore, the performance of Viola-Jones can be regarded as a conservative lower bound for the expected face detection performance of FaceReader^©^.

Table 4 presents the results obtained by applying both RetinaFace and the Viola-Jones Haar Cascade detector to every frame of the four evaluated datasets. As expected, RetinaFace achieved the best performance, failing to detect a face in only 100 frames out of more than 500,000 in CREMA-D. These failures predominantly corresponded to black transition frames located at the end of videos. Despite its simplicity, the Haar Cascade detector also achieved strong results, missing fewer than 0.02% and 0.2% of faces in RAVDESS and CREMA-D, respectively. Given these results, even under the conservative assumption that FaceReader^©^ relies on a face detector with performance comparable to or below that of Viola-Jones, face detection failures would be expected to have a negligible impact on the reported FER results.

**Table 4:** Face detection analysis.

Dataset	Number of faces	RetinaFaces' failures	Haar-Cascade's failures
WSEFEP	210	0	0
ADFES	216	0	0
RAVDESS	161,603	0	35
CREMA-D	564,785	100	1,153

### 4.2 Dataset results

#### 4.2.1 WSEFEP results

Table 5 presents the results obtained on the WSEFEP dataset. The highest performance was achieved by FaceReader^©^, with both WAR and UAR reaching 96.19%, followed by SwinFace and DAN-aff, whose values were approximately 10% lower. Among the evaluated VLMs, FaceLLM obtained the best results, achieving a WAR/UAR of 74.76%, however, despite being the strongest VLM, its performance remained comparable to that of the weakest traditional neural network, AP-ViT. In contrast, BLIP-2 exhibited the poorest behavior, with a WAR of 38.10%, performing more than 25% below the second-lowest model. Regarding class-specific behavior, *happiness* was consistently the most accurately classified emotion across all models, whereas *fear* was almost entirely unrecognized by many VLMs and *disgust* which was not detected a single time by PaliGemma-2 or BLIP-2.

**Table 5:** Results obtained with each model on the WSEFEP dataset.

WSEFEP Results (%)									
Model	Anger	Disgust	Fear	Happiness	Neutral	Sad	Surprise	WAR	UAR
DAN-raf	83.33	84.21	23.53	96.77	84.85	80.00	69.77	77.62 ± 04.19	77.62 ± 04.19
DAN-aff	89.23	95.24	81.08	100.0	86.79	83.33	66.67	86.67 ± 03.96	86.67 ± 03.96
SwinFace	86.57	89.29	71.70	100.0	90.91	86.67	86.21	87.62 ± 04.15	87.62 ± 04.15
ResMasknet	73.24	83.08	75.00	98.36	83.33	65.22	84.06	80.95 ± 04.76	80.95 ± 04.76
Ap-Vit	28.57	63.53	66.67	100.0	96.67	81.16	78.79	76.19 ± 04.24	76.19 ± 04.24
SmolVLM (Q3)	61.11	48.89	00.00	98.36	78.43	77.14	65.93	66.67 ± 04.40	66.67 ± 04.40
FaceLLM (Q1)	72.00	88.89	65.22	100.0	84.51	77.78	00.00	74.76 ± 03.66	74.76 ± 03.66
PaliGemma 1 (Q1)	46.81	79.25	00.00	100.0	80.00	80.00	66.67	70.48 ± 03.82	70.48 ± 03.82
PaliGemma 2 (Q1)	33.96	00.00	00.00	100.0	60.00	50.00	68.97	54.29 ± 03.47	54.29 ± 03.47
BLIP-2 (Q2)	28.75	00.00	00.00	100.0	55.32	43.33	06.06	38.09 ± 04.28	38.09 ± 04.28
FaceReader^©^ 10	94.92	95.08	93.10	100.0	96.77	98.31	95.08	**96.19** ± 02.55	**96.19** ± 02.55
Mean	63.50	66.13	43.30	99.41	81.60	74.81	62.56	73.59	73.59

The class-specific metric used is the F1-Score. In this database, the WAR and UAR are always equal, as the dataset is perfectly balanced. The highest results are highlighted in **bold**, and the second-best results are underlined. Q denotes the prompt used for each Vision-Language Model.

#### 4.2.2 ADFES results

The results achieved on the ADFES dataset are presented in Table 6. Similar to the WSEFEP dataset, FaceReader^©^ achieves the highest performance, with a WAR/UAR of 98.69%/98.67%, approximately 8% higher than the second-best model, DAN-aff. Overall, the results are comparable to those observed in WSEFEP, with FaceLLM being the best-performing VLM and achieving around 14% higher scores than SmolVLM, the second best. Despite this, FaceLLM results remained very similar to that achieved by AP-ViT, which remains the lowest-performing traditional neural network. Regarding class-specific performance, *surprise* shows improved recognition, with a mean F1-score of 75.08%, approximately 10 points higher than in WSEFEP. In contrast, *disgust* and *anger* exhibit lower scores, with mean F1-scores of 52.49% and 58.20%, respectively, roughly 10% and 6% worse than in WSEFEP. Meanwhile, *happiness* remains the best classified emotion, with almost every model achieving a perfect score on it. Regarding *fear* and *disgust*, the same trends seen on WSEFEP are repeated, as these emotions remained almost impossible to detect for the majority of the VLM models, with four different models achieving an F1-score of 0% for *fear* and three obtaining less than 10% in *disgust*.

**Table 6:** Results obtained with each model on the ADFES dataset.

ADFES results (%)									
Model	Anger	Disgust	Fear	Happiness	Neutral	Sad	Surprise	WAR	UAR
DAN-raf	70.00	76.60	70.59	100.0	91.30	90.91	82.35	83.66 ± 05.22	83.77 ± 05.18
DAN-aff	95.45	100.0	84.00	100.0	84.21	89.80	81.08	90.85 ± 04.16	90.72 ± 04.22
SwinFace	84.00	87.18	76.19	100.0	83.33	87.50	74.29	84.97 ± 05.27	84.82 ± 05.31
ResMasknet	91.30	89.80	75.68	97.78	92.68	85.00	87.50	88.89 ± 04.53	88.96 ± 04.50
Ap-Vit	00.00	64.29	85.00	100.0	90.00	77.19	88.89	76.47 ± 04.32	76.59 ± 04.31
SmolVLM (Q3)	50.85	16.67	00.00	100.0	90.91	79.17	61.54	64.05 ± 04.43	64.25 ± 04.42
FaceLLM (Q5)	59.38	08.00	77.78	100.0	97.78	97.67	85.71	78.43 ± 03.97	78.57 ± 03.94
PaliGemma 1 (Q1)	31.11	37.04	00.00	100.0	75.86	86.96	65.62	63.40 ± 04.14	63.64 ± 04.12
PaliGemma 2 (Q3)	05.00	00.00	00.00	100.0	67.69	74.51	67.74	55.56 ± 02.41	55.84 ± 02.40
BLIP-2 (Q4)	53.06	00.00	00.00	100.0	00.00	00.00	33.60	36.60 ± 02.95	37.01 ± 02.94
FaceReader^©^ 10	100.0	97.78	97.78	100.0	100.0	97.67	97.56	**98.69** ± 01.54	**98.67** ± 01.56
Mean	58.20	52.49	51.55	99.80	79.43	78.76	75.08	74.69	74.80

The class-specific metric used is the F1-Score. The highest results are highlighted in **bold**, and the second-best results are underlined. Q denotes the prompt used for each Vision-Language Model.

#### 4.2.3 RAVDESS results

The results obtained by each model on the RAVDESS dataset are presented in Table 7. Compared with the previously evaluated datasets, performance decreased substantially across all metrics. While the mean WAR and UAR on ADFES and WSEFEP were both approximately 74%, these values dropped to 44.96% and 46.49% on RAVDESS, representing a reduction of roughly 30% across all models. Although all models experienced a performance decline, FaceReader^©^ was affected the most, falling from consistently the best-performing model to outperforming only a single model. This commercial solution exhibited a decrease of approximately 60%, roughly double the average drop observed for the other models. Despite the overall lower scores and the pronounced decline of FaceReader^©^, other trends remained consistent: DAN trained on AffectNet remained the best-performing model overall, and BLIP-2 was the worst-performing model. It is worth noting that, in this case, FaceLLM did not perform best among the VLMs, achieving lower WAR and UAR than PaliGemma-1. Class-specific trends also persisted, with *happiness* achieving the highest scores and *anger* and *fear* the lowest while both, *fear* and *disgust*, were almost never correctly identified by the VLMs.

**Table 7:** Results obtained with each model on the RAVDESS dataset. The class-specific metric used is the F1-score.

RAVDESS results (%)									
Model	Anger	Disgust	Fear	Happiness	Neutral	Sad	Surprise	WAR	UAR
DAN-raf	19.09	67.23	13.59	74.69	41.92	50.58	43.06	50.16 ± 02.12	51.79 ± 02.26
DAN-aff	48.20	72.05	51.55	86.15	50.57	56.73	45.06	**60.50** ± 02.41	**61.16** ± 02.51
SwinFace	49.36	67.12	25.33	83.08	35.65	42.76	27.85	49.28 ± 02.21	52.83 ± 02.07
ResMasknet	49.50	67.29	23.42	81.33	40.95	42.07	44.98	52.88 ± 02.29	55.51 ± 02.26
Ap-Vit	10.84	51.25	10.78	72.18	39.55	54.71	18.03	44.95 ± 01.88	47.02 ± 02.06
SmolVLM (Q3)	24.69	37.55	00.00	65.50	38.75	43.61	38.46	40.78 ± 02.15	42.48 ± 02.32
FaceLLM (Q1)	06.06	68.65	47.78	90.78	28.49	36.63	07.80	44.07 ± 01.96	48.07 ± 01.82
PaliGemma 1 (Q3)	19.21	57.14	00.00	87.94	41.06	50.47	46.78	48.88 ± 02.17	50.00 ± 02.34
PaliGemma 2 (Q2)	24.52	00.00	04.00	83.67	00.00	36.74	34.93	36.78 ± 01.40	34.15 ± 01.30
BLIP-2 (Q2)	30.92	00.00	00.00	86.36	06.06	04.85	02.05	29.57 ± 00.89	27.68 ± 00.93
FaceReader^©^ 10	20.93	30.23	46.12	83.92	31.65	25.57	10.00	36.78 ± 02.15	40.70 ± 02.11
Mean	27.57	47.14	20.23	81.42	32.24	40.43	29.00	44.96	46.49

The highest results are highlighted in **bold**, and the second-best results are underlined. Q denotes the prompt used for each Vision-Language Model.

#### 4.2.4 CREMA-D results

The results obtained on the CREMA-D dataset are presented in Table 8. Overall, the mean performance of all models was the lowest among the evaluated datasets, with WAR and UAR of 36.09% and 36.55%, respectively. FaceReader^©^ experienced a substantial drop in performance again, achieving results below those of a random classifier, with WAR and UAR of only 14.89% and 15.88%. The highest performance was again achieved by DAN-aff, with WAR and UAR of 48.70% and 48.91%, followed by FaceLLM, which outperformed the other neural networks by a small margin. The relative improvement of FaceLLM can be attributed to the absence of the *surprise* emotion in CREMA-D, which in the RAVDESS and WSEFEP datasets was the category most detrimental to its performance. Class-specific trends remained consistent, with *happiness* continuing to be the most accurately recognized emotion across all models, while *anger* and *fear* exhibited the lowest recognition rates. Futhermore, the VLM models' tendency of missclassify *fear* and *disgust* remained visible in the results.

**Table 8:** Results obtained with each model on the CREMA-D dataset.

CREMA-D results (%)									
Model	Anger	Disgust	Fear	Happiness	Neutral	Sad	Surprise	WAR	UAR
DAN-raf	19.33	57.42	15.38	67.17	43.44	20.03	-	39.70 ± 00.88	40.23 ± 00.90
DAN-aff	32.39	67.54	41.42	76.18	46.98	37.41	-	**48.70** ± 01.07	**48.91** ± 01.07
SwinFace	30.10	44.81	14.88	80.85	43.64	24.10	-	42.56 ± 00.87	43.60 ± 00.87
ResMasknet	24.56	54.40	07.72	76.20	43.71	20.42	-	40.93 ± 00.90	41.62 ± 00.91
Ap-Vit	08.69	40.67	07.80	68.08	47.65	24.00	-	39.93 ± 00.81	40.41 ± 00.83
SmolVLM (Q3)	19.47	02.51	00.00	62.32	40.38	19.32	-	31.44 ± 00.70	32.32 ± 00.72
FaceLLM (Q1)	09.71	47.65	46.24	67.10	42.96	22.27	-	43.50 ± 00.89	44.22 ± 00.90
PaliGemma 1 (Q3)	06.23	47.67	00.16	77.94	43.59	30.83	-	35.78 ± 00.86	36.66 ± 00.87
PaliGemma 2 (Q2)	07.22	00.00	00.63	71.70	02.10	37.95	-	31.27 ± 00.48	30.55 ± 00.47
BLIP-2 (Q5)	31.99	00.00	00.00	73.16	00.00	00.00	-	28.32 ± 00.48	27.64 ± 00.47
FaceReader^©^ 10	00.79	03.89	14.37	27.43	22.92	01.39	-	14.89 ± 00.69	15.88 ± 00.72
Mean	17.32	33.32	13.51	65.01	34.01	21.61	-	36.09	36.55

The class-specific metric used is the F1-score. Note that the CREMA-D dataset has no *surprise* emotion. The highest results are highlighted in **bold**, and the second-best results are underlined. Q denotes the prompt used for each Vision-Language Model.

### 4.3 Discussion

The results obtained on the four datasets allow the drawing of several conclusions about the datasets and models tested, as certain aspects remain constistent in every experiment.

Regarding the datasets, it becomes clear that ADFES and WSEFEP constitute substantially easier conditions for the tested models in comparison with what RAVDESS and CREMA-D present, particularly the latter, which yields the lowest overall performance. In ADFES and WSEFEP, the average WAR/UAR of the evaluated models is approximately 74%/74%, whereas in RAVDESS it decreases to 44%/46% and in CREMA-D to 36%/36.5%, representing less than half the performance achieved in the more controlled datasets.

FaceLLM consistently achieves the best results among the tested VLMs in all but one dataset, which aligns with its intended purpose, as it is the only face-specialized VLM considered in this study. This observation is linked to a second conclusion that can be inferred from the results: all tested VLMs exhibit a pronounced bias toward the *happy* emotion. This class is consistently the best recognized, occasionally reaching an F1-score of 100%, while more negative emotions such as *fear, disgust*, or even *anger* are frequently almost invisible to the models, often obtaining an F1-score of 0%. This bias becomes even more accentuated in realistic conditions, as observed in CREMA-D, where this three classes have less than 10% of F1-score in three different models. In contrast, this bias is considerably reduced in FaceLLM, which demonstrates comparatively better performance on these negative emotions, often beign the only VLM capable of achieving comparable results to that of the traditional models, although the *surprise* class remains challenging, with results below 10% in both RAVDESS and WSEFEP datasets. Although this bias is also present in traditional models and in FaceReader^©^, the associated performance degradation is notably smaller.

Despite these observations regarding FaceLLM, its overall performance remains comparable to the worst-performing traditional neural network (AP-ViT), reinforcing the importance of task specialization in FER context, as happened with the VLMs. The best-performing traditional neural model, which is also the best model overall, is DAN trained on AffectNet. Although this model does not match the peak values obtained by FaceReader^©^ in ADFES and WSEFEP, it demonstrates significantly more stable and robust performance in RAVDESS and CREMA-D. From the performance gap observed between the DAN model trained on AffectNet and the version trained on RAF-DB, which amounts to approximately 10% in both metrics across all datasets, a clear difference in dataset generality can be inferred. These results indicate that AffectNet constitutes a more realistic and diverse scenario for FER tasks than RAF-DB, making it a more suitable choice for training models intended to generalize beyond controlled conditions.

Meanwhile, the overall worst-performing model is BLIP-2, which did not surpass 40% accuracy on any of the evaluated datasets and ranked last in all but one experimental setting, outperforming only FaceReader^©^ 10 on the CREMA-D dataset. One of the main issues observed with this model is the difficulty in obtaining a valid response. In many cases, particularly with the more challenging datasets, and despite the prompt optimization performed, the model either outputs “no” or “yes” in response to the prompt or consistenly predicts a single emotion, such as *happy* thereby making correct classification very difficult. Regarding the performance of others VLMs, it is also noticeable that PaliGemma 1 consistently outperforms PaliGemma 2 in this task, with differences ranging from nearly 16% on the simpler datasets to approximately 4% on CREMA-D.

Finally, with respect to the commercial FaceReader^©^ software, and consistent with the findings reported in previous works dicussed on Section 2, its performance appears optimal only under very controlled conditions, such as those found in ADFES and WSEFEP. In these datasets, the metrics reported by Noldus are largely reproduced, although a deviation of approximately 1% is observed in WSEFEP, and FaceReader^©^ achieves the best overall performance with a margin of roughly 10%. However, once the recording conditions become less constrained and the intensity of the displayed expressions decreases, its performance decreases substantially. In RAVDESS, FaceReader^©^ obtains a WAR/UAR of 36.75%/40.70%, and in CREMA-D its performance drops to 14.89%/15.88%, falling below the level expected from a random classifier.

### 4.4 Prompt results

As described in Section 3, five different prompts, previously reported in Table 2, were evaluated for each VLM across the four datasets. The corresponding results are presented in Table 9, organized by model and prompt. Overall, Q1, Q3, and Q4 yielded the best performance, corresponding to the three highest-scoring prompts for three of the five evaluated models. In contrast, these same prompts produced the poorest results for PaliGemma-2 and BLIP-2, which were also the lowest-performing models overall. These prompts provide the largest amount of contextual information and explicitly constrain the output to one of the basic emotions. Although this instruction was not always followed and a dictionary was required to map some responses to the target classes, the additional context appears to facilitate a better understanding of the task. Conversely, the best results for PaliGemma-2 and BLIP-2 were obtained with Q2 and Q5, which provide minimal context and directly request the emotion depicted or evoked in the image.

**Table 9:** Results obtained in terms of WAR and UAR in each dataset with the models and prompts tested.

Model	Prompt	WSEFEP	ADFES	RAVDESS	CREMA-D	Mean WAR	Mean UAR
SmolVML	Q1	59.52/59.52	57.52/57.67	39.90/**44.12**	29.82/**34.79**	46.69 ± 12.38	49.02 ± 10.14
	Q2	58.57/58.57	54.90/55.19	38.54/36.01	28.45/28.24	45.12 ± 12.23	44.50 ± 12.74
	Q3	**66.67**/**66.67**	**64.05**/**64.25**	**40.79**/42.49	**31.44**/32.32	**50.74** ± 15.02	**51.43** ± 14.51
	Q4	64.76/64.76	62.09/62.31	35.82/38.32	31.34/32.48	48.50 ± 15.04	49.47 ± 14.25
	Q5	57.14/57.14	52.94/53.25	38.30/35.71	28.77/28.71	44.29 ± 11.37	43.70 ± 11.84
FaceLLM	Q1	**74.76**/**74.76**	72.55/72.08	**44.07**/**48.07**	**43.50**/**44.22**	**58.72** ± 14.96	**59.78** ± 13.74
	Q2	62.38/62.38	67.97/68.18	31.89/36.76	33.85/35.27	49.02 ± 16.29	50.65 ± 14.79
	Q3	68.10/68.10	67.97/67.53	40.14/44.42	43.43/43.88	54.91 ± 13.17	55.98 ± 11.84
	Q4	68.10/68.10	69.93/69.48	42.63/42.86	40.67/39.92	55.33 ± 13.71	55.09 ± 13.75
	Q5	70.00/70.00	**78.43**/**78.57**	34.21/38.91	35.21/36.46	54.46 ± 19.98	55.99 ± 18.57
PaliGemma 1	Q1	**70.48**/**70.48**	**63.40**/**63.64**	30.13/35.12	25.25/26.92	47.31 ± 19.86	49.04 ± 18.41
	Q2	60.00/60.00	55.56/55.84	40.62/39.73	27.89/27.66	46.02 ± 12.69	45.81 ± 12.93
	Q3	63.81/63.81	60.13/60.39	**48.88**/**50.00**	**35.78**/**36.66**	**52.15** ± 10.93	**52.71** ± 10.57
	Q4	63.81/63.81	62.09/62.34	47.60/49.40	29.94/30.84	50.86 ± 13.62	51.60 ± 13.23
	Q5	56.67/56.67	54.90/55.19	37.26/34.67	30.38/29.69	44.80 ± 11.27	44.05 ± 12.02
PaliGemma 2	Q1	**54.29**/**54.29**	53.59/53.87	21.71/27.31	20.65/22.55	37.56 ± 16.39	39.50 ± 14.67
	Q2	50.95/50.95	51.63/51.95	**36.78**/**34.15**	**31.27**/**30.55**	**42.66** ± 08.85	41.90 ± 09.64
	Q3	53.33/53.33	**55.56**/**55.84**	30.93/35.57	25.00/26.75	41.20 ± 13.43	**42.87** ± 12.16
	Q4	53.33/53.33	**55.56**/**55.84**	26.20/31.18	23.87/25.69	39.74 ± 14.75	41.51 ± 13.25
	Q5	52.86/52.86	50.98/51.30	36.62/34.00	28.68/27.99	42.28 ± 10.06	41.54 ± 10.77
BLIP-2	Q1	18.57/18.57	19.61/19.88	06.97/08.71	00.64/00.73	11.45 ± 07.97	11.97 ± 07.80
	Q2	**38.10**/**38.10**	32.03/31.85	**29.57**/**27.68**	28.26/27.58	**31.99** ± 03.78	**31.30** ± 04.28
	Q3	14.29/14.29	14.38/14.29	15.38/14.29	17.08/16.67	15.28 ± 01.12	14.88 ± 01.03
	Q4	34.29/34.29	**36.60**/**37.01**	23.16/21.50	08.69/08.49	25.68 ± 11.05	25.32 ± 11.35
	Q5	28.57/28.57	28.76/28.57	28.04/26.04	**28.32**/**27.65**	28.42 ± 00.27	27.71 ± 01.03

The highest result for each model is in **bold**, and the second-best results are underlined.

The observed results highlight the importance of prompt engineering for VLM-based FER from two perspectives. First, significant performance differences can be obtained by applying different prompts to the same model. In most models, the gap between the best- and worst-performing prompts was approximately 8%, while for BLIP-2 this difference exceeded 20%, with Q1 achieving a WAR of 11.45% and Q2 reaching 31.99%. Second, the same prompt can produce markedly different results depending on the model. This behavior is exemplified by the set of prompts Q1, Q3, and Q4, which, as already discussed, yield the best results for three models while simultaneously producing the worst performance for two others. Large performance differences are observed even between models belonging to the same family, such as PaliGemma-1 and PaliGemma-2, further highlighting the model-dependent nature of prompt effectiveness.

## 5 Conclusion

This work presented a comprehensive evaluation of traditional neural networks, vision–language models, and the commercial software FaceReader^©^ 10 under a unified experimental protocol. Four widely benchmarked facial expression recognition (FER) datasets were employed, constrasting controlled conditions (ADFES and WSEFEP) with acted dynamic expressions with more realistic variability (RAVDESS and CREMA-D). Based on these evaluations, several conclusions can be drawn.

First, the WSEFEP and ADFES datasets, with mean WAR/UAR scores of 72.08%/72.08% and 72.49%/72.53%, respectively, were considerably easier for all evaluated models. In contrast, performance dropped sharply for the more realistic RAVDESS and CREMA-D datasets, which yielded 40.64%/44.12% and 32.56%/28.66%. This performance degradation of approximately 30%–40% confirms that evaluations on controlled static images substantially overestimate the true FER capabilities of all models and underscores the importance of using realistic, in-the-wild databases. In addition, all tested VLMs exhibited a pronounced bias toward *Happiness*, while negative emotions such as *Fear* and *Sadness* were frequently misclassified or entirely undetected. FaceLLM, the only VLM explicitly trained for biometric tasks, showed the most balanced behavior among the VLMs, yet its overall accuracy remained comparable to the weakest traditional model, highlighting the importance of domain-specific training.

Traditional FER networks demonstrated most robust generalization metrics, with DAN trained on AffectNet achieving the highest overall results. The consistent performance margin between DAN models trained on AffectNet vs. RAF-DB further indicates the greater suitability of AffectNet for training robust FER systems. The superior performance of models trained on domain-specific datasets reinforces the importance of training for this task.

Finally, FaceReader^©^ achieved excellent performance on controlled datasets, in agreement with its reported metrics, which were corroborated in this study. However, its performance deteriorated substantially in dynamic and less constrained scenarios, falling below the level of a random classifier in CREMA-D. These findings confirm an overspecialization of the software to controlled environments and highlight its need for improvement in more realistic operational conditions.

## Data Availability

The original contributions presented in the study are included in the article/supplementary material, further inquiries can be directed to the corresponding author.
